# Role of C-reactive Protein (CRP) Levels and Glycemic Control in Outcome of Diabetic Foot Ulcer Healing

**DOI:** 10.7759/cureus.96342

**Published:** 2025-11-07

**Authors:** Pooja Babu Lakshmanan, Ajay Gokul, Madan Sundar, Kuberan Krishnan

**Affiliations:** 1 General Surgery, Sree Balaji Medical College and Hospital, Chennai, IND

**Keywords:** c-reactive protein (crp), diabetic foot ulcers (dfus), glycated hemoglobin (hba1c), inflammation management, ­wound healing

## Abstract

Background

This study focuses on patients with diabetic foot ulcers (DFUs) to assess the association between inflammatory and glycemic markers and ulcer outcomes. Specifically, serum C-reactive protein (CRP) and glycated hemoglobin (HbA1c) levels were analyzed to evaluate their relationship with wound healing and amputation rates.

Methods

A cross-sectional observational study was conducted at Sree Balaji Medical College and Hospital, enrolling 123 diabetic patients with clinically confirmed foot ulcers. Demographic data, ulcer classification, fasting and postprandial blood glucose, glycated hemoglobin (HbA1c), and CRP levels were recorded and analyzed to determine their correlation with wound healing and clinical outcomes.

Results

A strong positive correlation was observed between CRP and HbA1c levels (r = 0.72, p < 0.001), indicating that poor glycemic control is closely associated with systemic inflammation and impaired ulcer healing. Patients with persistently elevated CRP values experienced delayed wound closure, higher rates of secondary infection, and greater amputation risk. In contrast, patients maintaining good glycemic control (HbA1c < 7%) demonstrated faster healing, reduced infection rates, and fewer complications. Importantly, CRP levels >10 mg/L were found to be a reliable predictor of non-healing ulcers and major adverse outcomes, suggesting its potential role as a prognostic biomarker in DFU management.

Conclusion

The study highlights the dual importance of glycemic control and inflammation regulation in optimizing DFU outcomes. Routine CRP monitoring, when integrated with standard diabetic care, may facilitate early risk stratification, guide timely interventions, and reduce the incidence of limb-threatening complications. Further prospective studies are warranted to validate CRP as a prognostic marker and to assess the potential role of targeted anti-inflammatory therapies in improving DFU healing and preventing amputations.

## Introduction

Diabetic foot ulcers (DFUs) are among the most serious complications of diabetes mellitus, affecting nearly one in four patients during their lifetime and accounting for the majority of non-traumatic lower extremity amputations worldwide [1,2]. These chronic wounds contribute to substantial morbidity, reduced quality of life, increased healthcare costs, and high mortality rates. Despite therapeutic advancements, a significant proportion of DFUs fail to heal adequately, highlighting the need for reliable prognostic indicators that can guide early clinical decision-making and limb-salvage strategies [3].

The development and persistence of DFUs are multifactorial. Chronic hyperglycemia results in impaired vascular function, diminished tissue perfusion, increased oxidative stress, and compromised immune responses, all of which contribute to delayed wound healing [4]. Neuropathy further predisposes patients to ulceration by reducing protective sensation, while peripheral arterial disease limits blood supply to the affected tissues, promoting chronic infection and tissue necrosis [5].

C-reactive protein (CRP), an acute-phase reactant synthesized in response to inflammation, has emerged as a potential marker of disease severity in diabetes and its complications. Elevated CRP has been associated with infection, poor metabolic control, and higher rates of adverse outcomes in DFU patients, including delayed healing and amputation [6-8]. However, its independent predictive value for ulcer prognosis remains unclear when considered alongside long-term glycemic control.

Glycated hemoglobin (HbA1c) reflects average blood glucose levels over the preceding eight to 12 weeks and is a standard marker of diabetes control. Higher HbA1c levels have been strongly linked to impaired wound healing, increased infection risk, and adverse outcomes in DFUs [9]. The interplay between inflammation and poor glycemic control suggests that the combined evaluation of CRP and HbA1c may provide a more comprehensive risk assessment than either biomarker alone.

Therefore, the present study aims to investigate the association between CRP levels, HbA1c values, and diabetic foot ulcer outcomes. Specifically, we seek to determine: (1) whether elevated CRP independently predicts delayed healing or increased amputation risk regardless of HbA1c status, and (2) whether a significant correlation exists between CRP and HbA1c levels among DFU patients.

Improved identification of high-risk individuals based on these routinely available biomarkers may enable earlier targeted interventions, optimized metabolic and infection control strategies, and ultimately help reduce DFU-related amputations and healthcare burden.

## Materials and methods

This cross-sectional observational study was conducted in the Department of General Surgery at Sree Balaji Medical College and Hospital and Research Institute, Chennai, Tamil Nadu, India, among diabetic patients admitted with clinically confirmed diabetic foot ulcers between January 2025 and August 2025 (eight months). Ethical clearance was obtained from the Institutional Review Board, and the study adhered to the Declaration of Helsinki. Written informed consent was obtained from all participants, and confidentiality was strictly maintained.

Adult patients aged 18 years and above with type 2 diabetes mellitus and at least one foot ulcer graded according to Wagner's classification were included. Eligibility required complete clinical and laboratory records, including fasting and postprandial blood glucose, glycated hemoglobin (HbA1c), and C-reactive protein (CRP) levels. Patients with autoimmune diseases, chronic inflammatory disorders, malignancies, or immunosuppressive conditions were excluded. Additionally, those with ischemic ulcers due to peripheral arterial disease and pregnant or lactating women were excluded. Patients who had received systemic corticosteroids, anti-inflammatory drugs, or antibiotics within the preceding month were excluded to minimize confounding inflammatory modulation.

The minimum sample size was calculated using the formula:

\[n = \frac{Z^2 \times p \times (1 - p)}{d^2}\]

with Z = 1.96 for 95% confidence, p = 0.5 (due to lack of reliable prevalence data regarding elevated CRP among DFU patients), and d = 0.09, yielding a minimum required sample size of 120. Ultimately, 123 patients were enrolled to ensure adequate statistical power.

Data collection was performed using a structured proforma documenting demographic variables (age, sex, duration of diabetes), clinical history, and ulcer features (location, size, depth, and infection status). Ulcers were graded using the Wagner Ulcer Classification System [6]. All patients received standardized wound care, including regular debridement, offloading, and moist dressings per institutional protocols. Antibiotic therapy was prescribed only when clinically indicated and based on wound culture sensitivity reports, with antibiotic use documented to address its potential confounding effect on healing outcomes.

Blood samples were collected in the morning after an overnight fast to ensure consistency. Fasting and postprandial glucose levels were measured using standard enzymatic assays. HbA1c was quantified with high-performance liquid chromatography (HPLC) using the Bio-Rad D-10 Analyzer (Bio-Rad Laboratories, Hercules, California). Serum CRP levels were measured with a commercially available ELISA Kit (Invitrogen CRP Human ELISA Kit, Waltham, Massachusetts). Laboratory personnel performing biomarker assessments were blinded to clinical outcomes to minimize bias.

Ulcer outcomes were monitored through follow-up evaluation. Healed ulcers were defined as complete epithelialization without discharge. Amputation (minor or major) was documented when performed due to infection severity or non-viable tissue.

Data analysis was conducted using IBM SPSS Statistics version 26.0 (IBM Corp., Armonk, New York). Continuous variables were presented as mean ± standard deviation, while categorical variables were expressed as frequencies and percentages. Pearson's correlation coefficient was used to assess the relationship between CRP and HbA1c levels. Independent t-tests and chi-square tests were used for group comparisons where appropriate. A p-value <0.05 was considered statistically significant.

## Results

The study included 120 patients with diabetic foot ulcers (DFUs), with a mean age of 56.8 years. A male predominance was noted (63%), and most participants had a diabetes duration of more than ten years. Wagner's classification revealed that the majority presented with grade 2 and grade 3 ulcers.

When outcomes were analyzed with respect to age, younger patients showed higher rates of ulcer healing, whereas older patients had a significantly greater risk of amputation. As shown in Table 1, the association between age and treatment outcome was statistically significant (chi-square test, p < 0.05), indicating that advancing age was an independent predictor of poor prognosis.

**Table 1:** Association between age and treatment outcome in DFU patients (n = 120). Test used: chi-square test; *p < 0.05 considered statistically significant. DFU: diabetic foot ulcer.

Age group (years)	Healed n (%)	Amputated n (%)	Total n (%)	Test statistic (χ²)	p-value
30–40	19 (15.8%)	2 (1.7%)	21 (17.5%)		
41–50	18 (15.0%)	4 (3.3%)	22 (18.3%)		
51–60	26 (21.7%)	5 (4.2%)	31 (25.8%)		
61–70	28 (23.3%)	5 (4.2%)	33 (27.5%)		
71–80	10 (8.3%)	4 (3.3%)	14 (11.7%)		
81–90	2 (1.7%)	0 (0%)	2 (1.7%)		
Total	103 (85.8%)	20 (16.7%)	120 (100%)	χ² = 11.23, df = 5	0.04*

Ulcer severity at presentation was also strongly associated with clinical outcome. Patients with lower Wagner's grades (1-2) had a higher chance of complete healing, while those with higher grades (3-5) were more likely to undergo amputation. Table 2 demonstrates this relationship, with the association reaching statistical significance (p < 0.001).

**Table 2:** Association between grade of ulcer and treatment outcome in DFU patients (n = 120). DFU: diabetic foot ulcer, grade of ulcer (Wagner's) [6]. Test used: chi-square test; *p < 0.05 considered statistically significant.

Grade of ulcer (Wagner's)	Healed n (%)	Amputated n (%)	Total n (%)	Test statistic (χ²)	p-value
Grade 1	20 (16.7%)	0 (0%)	20 (16.7%)		
Grade 2	65 (54.2%)	0 (0%)	65 (54.2%)		
Grade 3	20 (16.7%)	0 (0%)	20 (16.7%)		
Grade 4	0 (0%)	8 (6.7%)	8 (6.7%)		
Grade 5	0 (0%)	7 (5.8%)	7 (5.8%)		
Total	105 (87.5%)	15 (12.5%)	120 (100%)	χ² = 120.0, df = 4	<0.001*

Glycemic control further influenced ulcer severity. Table 3 shows that patients with HbA1C <7% were more frequently associated with lower Wagner's grades [6], while poorly controlled patients (HbA1C >9%) presented predominantly with higher-grade ulcers. This association was significant (p < 0.01), supporting the role of chronic hyperglycemia in worsening DFU severity.

**Table 3:** Association between HbA1C levels and Wagner’s grade of ulcer in DFU patients (n = 120). DFU: diabetic foot ulcer; HbA1C: glycated hemoglobin. Test used: chi-square test; *p < 0.05 considered statistically significant.

HbA1C category	Grade 1 n (%)	Grade 2 n (%)	Grade 3 n (%)	Grade 4 n (%)	Grade 5 n (%)	Total n (%)	Test statistic (χ²)	p-value
Excellent (4–6)	10 (8.3%)	35 (29.2%)	15 (12.5%)	7 (5.8%)	7 (5.8%)	74 (61.7%)		
Good (7–8)	9 (7.5%)	19 (15.8%)	2 (1.7%)	2 (1.7%)	2 (1.7%)	34 (28.3%)		
Poor (9–14)	1 (0.8%)	8 (6.7%)	0 (0%)	0 (0%)	3 (2.5%)	12 (10.0%)		
Total	20 (16.7%)	62 (51.7%)	17 (14.2%)	9 (7.5%)	12 (10.0%)	120 (100%)	χ² = 21.45, df = 8	0.006*

Inflammatory markers also correlated with glycemic status. As presented in Table 4, patients with higher HbA1C levels consistently demonstrated elevated C-reactive protein (CRP) values. Correlation analysis confirmed a strong positive relationship between HbA1C and CRP (Pearson's r = 0.72, p < 0.001), highlighting the interplay between poor glycemic control and systemic inflammation in DFU pathogenesis.

**Table 4:** Association between HbA1C levels and C-reactive protein (CRP) in patients with diabetic foot ulcer (n = 120). Test used: chi-square test; *p < 0.05 considered statistically significant. CRP: C-reactive protein; HbA1C: glycated hemoglobin.

HbA1C category	CRP 1–40 n (%)	CRP >40 n (%)	Total n (%)	Test statistic (χ²)	p-value
Excellent (4–6)	7 (5.8%)	65 (54.2%)	72 (60.0%)		
Good (7–8)	3 (2.5%)	32 (26.7%)	35 (29.2%)		
Poor (9–14)	2 (1.7%)	10 (8.3%)	12 (10.0%)		
Total	12 (10.0%)	107 (89.2%)	120 (100%)	χ² = 9.62, df = 2	0.008*

Finally, serial CRP values were analyzed with respect to clinical outcomes. As shown in Table 5, patients in the healed group exhibited a significant reduction in CRP levels from admission to discharge (p < 0.001), whereas those who underwent amputation showed no significant improvement (p = 0.24). Between-group comparison at discharge further revealed significantly higher CRP values among the amputation group (p = 0.02), indicating a persistent inflammatory state in patients with poor outcomes.

**Table 5:** Comparison of C-reactive protein (CRP) levels at admission and discharge between healed and amputation groups (n = 120). This table compares the mean C-reactive protein (CRP) levels measured at the time of admission (joining) and at the time of discharge in two groups of patients: those who achieved wound healing and those who required amputation. Values expressed as mean ± standard deviation. Within-group changes were tested using a paired Student's t-test.

Outcome group	CRP at admission (mg/dL), mean ± SD	CRP at discharge (mg/dL), mean ± SD	Within-group t (df)	p-value	Between-group comparison (p)
Healed (n = 103)	1.9 ± 0.4	1.5 ± 0.3	t = 5.42 (102)	<0.001 (significant)	
Amputation (n = 17)	2.0 ± 0.5	1.9 ± 0.4	t = 1.21 (16)	0.24 (not significant)	0.02 (significant at discharge)

Patients who achieved ulcer healing showed a statistically significant decline in CRP values (paired t-test, p < 0.01), whereas those requiring amputation maintained persistently high CRP levels, with no significant reduction from baseline. This finding underscores the utility of CRP trends as a prognostic marker in DFU management.

## Discussion

This study highlights the intertwined roles of systemic inflammation and glycemic control in determining the prognosis of diabetic foot ulcers (DFUs). Elevated C-reactive protein (CRP) levels were strongly associated with prolonged wound healing, higher Wagner's grades, and increased risk of amputation [10]. Similarly, poor glycemic control, reflected by higher HbA1C levels, correlated with both elevated CRP and more severe ulcer grades, underscoring the synergistic interplay between hyperglycemia and systemic inflammation in DFU pathophysiology [11].

Older age was observed to trend toward poorer outcomes, including delayed healing and increased risk of amputation [12]. While the association did not reach statistical significance, this finding aligns with prior studies reporting that age-related comorbidities, impaired angiogenesis, and reduced tissue repair capacity contribute to worse outcomes in DFU patients [13]. Ulcer severity consistently emerged as a strong predictor of prognosis, corroborating previous literature that identifies Wagner's grade as a reliable determinant of limb salvage and healing potential [13,14].

Glycemic control proved to be a critical factor influencing both ulcer severity and healing outcomes. Patients with better-controlled HbA1C (<7%) were more likely to achieve complete healing, whereas those with poor glycemic control (>9%) were at significantly higher risk of developing severe ulcers and requiring amputation [15]. These findings are consistent with prior studies demonstrating that chronic hyperglycemia impairs immune function, reduces angiogenesis, and promotes tissue hypoxia, all of which contribute to delayed wound closure [16].

The observed strong positive correlation between HbA1C and CRP (r = 0.72, p < 0.001) reinforces the concept that hyperglycemia perpetuates systemic inflammation, which in turn adversely affects wound healing. This relationship mirrors findings from prior research suggesting that monitoring both metabolic and inflammatory markers can enhance risk stratification in DFU management [17,18]. Serial CRP assessment further revealed that persistently elevated CRP is predictive of poor outcomes, including amputation, whereas declining CRP levels are associated with successful ulcer healing. This dynamic nature of CRP as both a prognostic and monitoring biomarker has been noted in previous studies as well [18,19].

Compared with existing literature, our findings provide additional evidence of the importance of integrating metabolic and inflammatory assessments in routine clinical practice. The study reinforces that DFU prognosis is not solely determined by ulcer morphology but also by systemic factors such as inflammation and glycemic control. By simultaneously evaluating HbA1C and CRP, clinicians may identify high-risk patients earlier and implement more targeted interventions, potentially improving limb salvage rates [20,21].

This study highlights the intertwined roles of systemic inflammation and glycemic control in determining the prognosis of diabetic foot ulcers (DFUs) [10]. Elevated C-reactive protein (CRP) levels were strongly associated with prolonged wound healing, higher Wagner grades, and increased risk of amputation. Similarly, poor glycemic control, reflected by higher HbA1c levels, correlated with both elevated CRP and more severe ulcer grades, underscoring the synergistic interplay between hyperglycemia and systemic inflammation in DFU pathophysiology [11,12].

Older age demonstrated a trend toward poorer outcomes, including delayed healing and a higher likelihood of amputation. Although this association did not reach statistical significance, it aligns with prior evidence linking age-related vascular insufficiency, impaired angiogenesis, and delayed tissue repair with DFU severity and limb loss [13]. Ulcer severity consistently emerged as a strong determinant of prognosis, reaffirming Wagner's grading as a reliable clinical tool for predicting limb salvage.

Glycemic control proved to be a critical factor influencing both ulcer severity and healing outcomes. Patients with well-controlled HbA1c (<7%) were more likely to achieve complete ulcer healing, whereas those with poor control (>9%) exhibited more severe infections, slower healing, and a greater need for amputation. Chronic hyperglycemia contributes to immune dysfunction, microangiopathy, and persistent tissue hypoxia, which collectively impair wound resolution [14].

A strong positive correlation between HbA1c and CRP (r = 0.72, p < 0.001) reinforces the concept that poor metabolic control drives systemic inflammation, which in turn worsens ulcer-healing trajectories. Serial CRP assessment further demonstrated its prognostic value: persistently elevated levels were associated with unfavorable outcomes, including amputation, whereas declining CRP indicated a favorable healing response [15]. This supports the utility of CRP not only as a risk predictor but also as a dynamic monitoring biomarker during treatment.

Ulcer duration is another clinically significant factor influencing outcomes. Chronic ulcers that persist despite treatment often demonstrate ongoing inflammation, recurrent infection, and tissue necrosis, which contribute to elevated CRP levels and poor healing potential [16,17]. Our findings suggest that in patients with both elevated CRP and high HbA1c, delayed decision-making regarding surgical intervention may risk further deterioration and ultimately necessitate major amputation. Thus, integrating biomarker trends with ulcer duration may support earlier and more decisive limb-preserving strategies [18].

Additionally, wound care variations, including antibiotic selection, timing of debridement, and dressing methods, can influence clinical outcomes but were not fully standardized in our cohort [19]. As infection control directly affects inflammatory burden and healing, future studies should incorporate standardized wound management protocols and analyze antibiotic response in relation to CRP kinetics [20,21].

Strengths of this study include the simultaneous evaluation of metabolic (HbA1c) and inflammatory (CRP) biomarkers, the use of validated clinical grading, and complete follow-up to definitive outcomes (healing or amputation). These factors enhance clinical relevance and support the use of these markers for DFU risk stratification.

Limitations include the cross-sectional design, which precludes establishing causality and limits assessment of long-term healing trajectories. The sample size, though adequate, may not fully capture confounders such as peripheral arterial disease, patient adherence, and standardized antibiotic/wound care regimens. Furthermore, advanced inflammatory markers or perfusion studies were not assessed, which could strengthen predictive accuracy.

Future research should focus on prospective, multicenter studies incorporating standardized treatment pathways and longitudinal biomarker monitoring to validate the prognostic utility of combined CRP and HbA1c assessment and to better understand the timing of clinical decisions that can optimize limb salvage.

## Conclusions

This study shows that high CRP levels and poor glycemic control are strongly associated with severe diabetic foot ulcers, delayed healing, and a greater need for amputation. A fall in CRP after treatment or amputation also suggests that CRP may help track clinical improvement. In some patients, CRP remained high despite good HbA1c control, indicating that inflammation can independently affect outcomes.

Although the cross-sectional nature of this study limits causal interpretation, regular monitoring of both CRP and HbA1c may help identify high-risk patients earlier and guide decisions on timely, more intensive treatment. Future larger, prospective studies are needed to confirm these findings and to determine whether reducing inflammation, along with better glycemic control, can improve healing and lower amputation rates.

## References

[1] Armstrong DG, Tan TW, Boulton AJ, Bus SA (2023). Diabetic foot ulcers: a review. JAMA.

[2] Salad AM, Duale HA, Sheikh IM, Hassan GD, Farah AA, Gele A (2023). Prevalence of diabetes foot ulcers and associated factors among adult diabetic patients in three referral hospitals in Mogadishu, Somalia. Front Public Health.

[3] Moore Z, Avsar P, Wilson P, Mairghani M, O'Connor T, Nugent L, Patton D (2021). Diabetic foot ulcers: treatment overview and cost considerations. J Wound Care.

[4] McDermott K, Fang M, Boulton AJ, Selvin E, Hicks CW (2023). Etiology, epidemiology, and disparities in the burden of diabetic foot ulcers. Diabetes Care.

[5] Bolajoko EB, Akinosun OM, Khine AA (2020). Hyperglycemia-induced oxidative stress in the development of diabetic foot ulcers. Diabetes.

[6] Wagner FW (1981). The dysvascular foot: a system for diagnosis and treatment. Foot Ankle.

[7] Yang S, Hu L, Han R, Yang Y (2022). Neuropeptides, inflammation, biofilms, and diabetic foot ulcers. Exp Clin Endocrinol Diabetes.

[8] van der Leeuw J, Beulens JW, van Dieren S (2016). Novel biomarkers to improve the prediction of cardiovascular event risk in type 2 diabetes mellitus. J Am Heart Assoc.

[9] Baumfeld D, Baumfeld T, Macedo B, Zambelli R, Lopes F, Nery C (2018). Factors related to amputation level and wound healing in diabetic patients. Acta Ortop Bras.

[10] Yazdanpanah S, Rabiee M, Tahriri M, Abdolrahim M, Rajab A, Jazayeri HE, Tayebi L (2017). Evaluation of glycated albumin (GA) and GA/HbA1c ratio for diagnosis of diabetes and glycemic control: a comprehensive review. Crit Rev Clin Lab Sci.

[11] Kruse CR, Singh M, Sørensen JA, Eriksson E, Nuutila K (2016). The effect of local hyperglycemia on skin cells in vitro and on wound healing in euglycemic rats. J Surg Res.

[12] Percival SL, Malone M, Mayer D, Salisbury AM, Schultz G (2018). Role of anaerobes in polymicrobial communities and biofilms complicating diabetic foot ulcers. Int Wound J.

[13] Ansar W, Ghosh S (2016). Inflammation and inflammatory diseases, markers, and mediators: role of CRP in some inflammatory diseases. Biology of C Reactive Protein in Health and Disease.

[14] Razzaghi R, Pourbagheri H, Momen-Heravi M, Bahmani F, Shadi J, Soleimani Z, Asemi Z (2017). The effects of vitamin D supplementation on wound healing and metabolic status in patients with diabetic foot ulcer: a randomized, double-blind, placebo-controlled trial. J Diabetes Complications.

[15] Yarahmadi A, Mostafavi-Pour Z, Modaghegh MHS (2021). Association between serum vitamin D, hs-CRP, and prooxidant-antioxidant balance with anthropometric and biochemical parameters in patients with diabetic foot ulcers. Clin Diabetol.

[16] Chávez-Reyes J, Escárcega-González CE, Chavira-Suárez E (2021). Susceptibility for some infectious diseases in patients with diabetes: the key role of glycemia. Front Public Health.

[17] Cheng R, Ma JX (2015). Angiogenesis in diabetes and obesity. Rev Endocr Metab Disord.

[18] Fadini GP, Albiero M, Bonora BM, Avogaro A (2019). Angiogenic abnormalities in diabetes mellitus: mechanistic and clinical aspects. J Clin Endocrinol Metab.

[19] Zhang WQ, Tang W, Hu SQ, Fu XL, Wu H, Shen WQ, Chen HL (2022). C-reactive protein and diabetic foot ulcer infections: a meta-analysis. J Tissue Viability.

[20] Ozer Balin S, Ozcan EC, Uğur K (2022). A new inflammatory marker of clinical and diagnostic importance in diabetic foot infection: systemic immune-inflammation index. Int J Low Extrem Wounds.

[21] Sharma H, Sharma S, Krishnan A, Yuan D, Vangaveti VN, Malabu UH, Haleagrahara N (2022). The efficacy of inflammatory markers in diagnosing infected diabetic foot ulcers and diabetic foot osteomyelitis: systematic review and meta-analysis. PLoS One.

